# Design and Evaluation of Novel Textile Wearable Systems for the Surveillance of Vital Signals

**DOI:** 10.3390/s16101573

**Published:** 2016-09-24

**Authors:** Isabel G. Trindade, José Machado da Silva, Rui Miguel, Madalena Pereira, José Lucas, Luís Oliveira, Bruno Valentim, Jorge Barreto, Manuel Santos Silva

**Affiliations:** 1 IBEB-FCUL, College of Sciences of University of Lisbon, 1749-016 Lisbon, Portugal; 2 FibEnTech Unit, University of Beira Interior, 6201-001 Covilhã, Portugal; rmiguel@ubi.pt (R.M.); mmrp@ubi.pt (M.P.); jlucas@ubi.pt (J.L.); mjssilva@ubi.pt (M.S.S.); 3 INESC TEC, Faculty of Engineering of University of Porto, 4200-465 Porto, Portugal; jms@fe.up.pt; 4 CHCB, Hospital Centre of Cova da Beira, 6200-251 Covilhã, Portugal; luisvoliveira@sapo.pt (L.O.); bruno.valentim81@gmail.com (B.V.); 5 Playvest S.A., Rua das Austrálias, 2, 4705-322 Braga, Portugal; jorgebarreto@playvest.pt

**Keywords:** textile wearable technologies, flexible electronics, mHealth, pHealth

## Abstract

This article addresses the design, development, and evaluation of T-shirt prototypes that embed novel textile sensors for the capture of cardio and respiratory signals. The sensors are connected through textile interconnects to either an embedded custom-designed data acquisition and transmission unit or to snap fastener terminals for connection to external monitoring devices. The performance of the T-shirt prototype is evaluated in terms of signal-to-noise ratio amplitude and signal interference caused by baseline wander and motion artefacts, through laboratory tests with subjects in standing and walking conditions. Performance tests were also conducted in a hospital environment using a T-shirt prototype connected to a commercial three-channel Holter monitoring device. The textile sensors and interconnects were realized with the assistance of an industrial six-needle digital embroidery tool and their resistance to wear addressed with normalized tests of laundering and abrasion. The performance of these wearable systems is discussed, and pathways and methods for their optimization are highlighted.

## 1. Introduction

Recent progress in mobile and wearable technologies enabled by low-power electronics, new signal processing techniques [1] and, in general, the information and communication technologies (ICT) ecosystem, open possibilities for a new paradigm in medicine and healthcare. There is an on-going trend in the healthcare systems to replace the old hospital-centric model with a preventive patient-centric model [2]. In the early 2000s, smart textile wearable technologies were considered strategic to lower morbidity and healthcare costs associated to chronic diseases of the circulatory system, leading to the development of garments with embedded sensors connected to personal digital assistant (PDA) portable devices [3,4,5,6,7,8,9]. More recently, wearable textile technologies have been further considered to prevent illness and promote wellbeing and active aging, for an effective reduction of physical and mental decline with age, and a sustainable healthcare system [10]. These goals can be achieved within the strategies defined for mobile and personalized health (mHealth and pHealth), with several challenges to be addressed, such as the energy autonomy of portable electronics [1,11], specific design aspects of the wearables, particularly regarding comfort, perception, and interface to the users, the captured data quality, as well as data privacy and security [10].

Clothing and apparel, having a large portable surface and the possibility of integrating built-in sensors, pockets, and embedded electronics, have great potential for application in wearable medical systems [12,13]. The use of conventional standard gel electrodes puts several limitations on the users; they are not re-usable, quickly degrade over time as the gel dries out, the gel often causes skin irritation and the growth of undesired bacteria, and are perceived by the user as uncomfortable. Dry electrodes have been proposed as a viable alternative, enabling unobtrusive placement of the electrodes on specified regions of the wearer’s body, while providing accurate monitoring of the physiological signals, such as electrocardiogram (ECG), electromyography (EMG) [14], pulmonary and abdominal respiration [15,16], electrodermal impedance, and galvanic skin response [17]. Resorting to embedded textile conductors to interconnect different data acquisition nodes, detailed comprehensive information on the wearer’s health status can be obtained. Multiple sensors help cut down computational processing and allow for data fusion to obtain more reliable information. Several technologies and approaches have been pursued to realize dry electrodes; knitted electrodes manufactured by seamless knitting technology and the method of intarsia [18,19]; woven electrodes produced by Jacquard technology [20,21]; polymeric removable electrodes with snap fastener connectors for attachment to the garment [22]; conductive foam electrodes integrated in a T-shirt with electrically-conductive ink-printed interconnects [23]; and embroidery stitching of electrodes [16,24,25,26]. 

In our previous work [16] we presented a chest-band system to capture cardiorespiratory signals, embedding three in-line embroidery electrodes and two capacitive electrodes, realized with the assistance of an embroidery method. A study of the signal-to-noise ratio (SNR) of the embroidery electrodes under dry and moist conditions, realized with several subjects, was also presented [27], indicating that the presence of moisture in the textile electrodes is often necessary to attain SNR amplitudes similar to those obtained with gel electrodes. Effectively, those experimental tests showed that, over time, physical contact between dry electrodes and the skin causes the absorption of sweat/moisture by the electrodes, as indicated by the stabilization of an enhanced SNR after 10 to 30 min of the initiation of the tests, to amplitudes similar to those attained with gel and dry textile electrodes that were moist in the initial test condition. In the work presented here, the embroidery method was applied to assist the realization of T-shirt systems for the surveillance of vital signals. Three prototypes are presented; one, of first generation, with cardiorespiratory sensing functions, was meant for laboratory tests and provided insights that led to major improvements in the performance of the second generation T-shirt systems. The two second generation prototypes comprise cardio-sensing functions and use distinct concepts of integration. The novel T-shirt design, for a patient-centric model, embeds a custom data acquisition and transmission (DAT) unit with a low-energy wireless Bluetooth link to a smartphone. The smartphone runs an app that receives, records, and displays in real-time the wearer’s ECG signals. Another prototype, embedding analogous textile electrodes and interconnects, and integrating snap fastener terminals for external connection to a Holter monitoring device was also realized and evaluated with five patients in a hospital environment. 

The rest of the article is organized as follows: in Section 2 we describe the design and materials of the T-shirt systems and the experimental methods used to evaluate their performance in laboratory and hospital settings, and to address their resistance to wear; in Section 3 we present the custom DAT unit and the studies and methods carried out to reduce the power consumption involved in the signal processing operations; experimental results and discussion are presented in Section 4; and conclusions are highlighted in Section 5. 

## 2. Materials and Methods

### 2.1. T-Shirt Design, Construction, and Sensor Integration

Three T-shirt prototypes were realized. A prototype for cardiorespiratory surveillance that used first generation tubular knits with homogeneous structures and two cardio surveillance prototypes, using second generation T-shirt bodies, integrating localized compression bands in the tubular knits. The T-shirts were designed in order to be elastic and had a conformal, tight fit to the wearer’s body, and manufactured from seamless tubular knits produced with circular looms, having a composition of 92% polyamide and 8% elastane [28].

**Figure 1:**
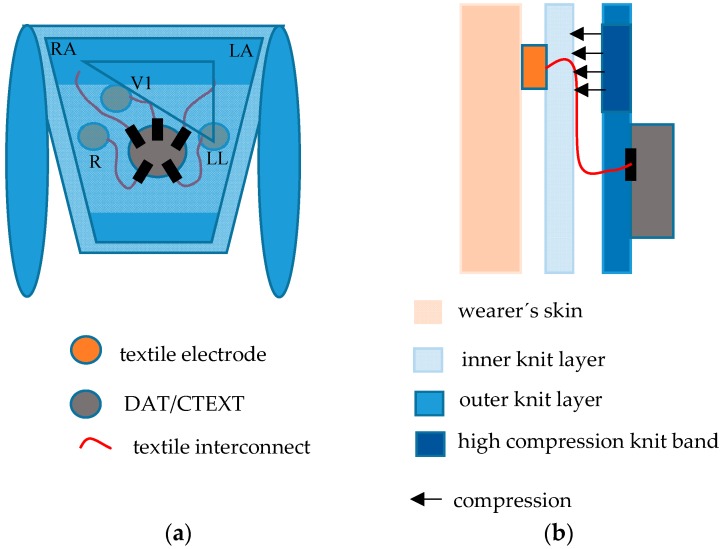
Schematics of T-shirt embedding textile sensors, textile interconnectors and either a custom DAT unit or terminals for electrical connection to an external data acquisition unit; (**a**) front-view representation; and (**b**) cross-section representation.

The generic design of the prototypes is schematically described in Figure 1. The body of the T-shirt consists of two juxtaposed (double-layer) tubular knit fabrics. The inner knit layer, in direct contact to the wearer’s skin, provides the support for the sensors. The outer layer integrates either a custom DAT or terminals for connection to an external data acquisition unit (CTEXT). The knit fabrics embed textile cables to interconnect the sensors to the DAT/CTEXT. The T-shirts comprise five skin-contact textile electrodes to capture the ECG signals and can also integrate, in the external side of the inner knit layer, two textile capacitive sensors to capture respiratory signals. The outer knit layer applies pressure over the sensing regions for good electrical electrode-skin contact. The cardiac signals are captured with the five-lead configuration typically used in ambulatory exams. The wearable textile system places five embedded skin-contact textile electrodes in the wearer’s thorax region; three electrodes in the limbs region, corresponding to the triangle of Einthoven and Wilson (EW) to obtain the derivations, lead 1, 2, 3 (L1, L2, L3); the fourth and reference electrodes (R) in the regions of the precordial V1, and either above the sternum or opposite to the lower limb apex of the EW, respectively. 

The second generation prototypes are composed of heterogeneous knit structures, integrating bands of compression to enhance the applied pressure over the sensing regions. The bands were incorporated through suitable adjustment of the structure and density in the knit construction. In contrast, in the region covering the user’s abdomen, the knit fabrics exhibit considerable elasticity to fit wearers with physical conditions classified as overweight or obese. 

**Figure 2:**
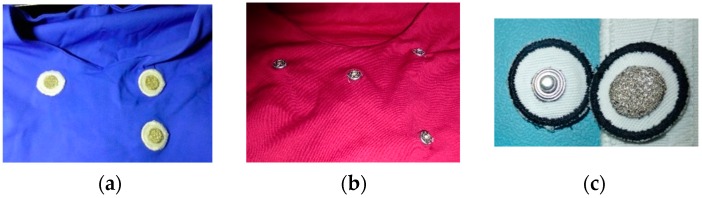
Photos showing partial views of inner layer from two versions of T-shirts; (**a**) with non-removable embroidery electrodes; (**b**) with snap fasteners; and (**c**) a photo of removable embroidery electrodes.

Prototypes using different methods of integration of ECG sensors were used. In one prototype, the inner knit layer embedded five non-removable embroidery electrodes, while, in the other prototype, snap fastener terminals (SFC) were integrated for attachment of removable embroidery electrodes. Figure 2 shows the two types of integrations and photos of the removable embroidery electrodes. The embroidery electrodes were designed to be washable and long-lasting, comfortable to wear in contact to the skin, and inexpensive [16]. They consist of electrically-conductive embroidery patterns, realized with commercial conductive threads, having a circular shape (16 mm Ø). The removable dry electrodes have on their back side a male snap fastener, electrically connected to the electrode in the front side of the sensor. The capacitive sensors consist of commercial conductive woven fabrics with a rectangular shape (5 cm × 4 cm) realized also with the assistance of the embroidery stitching method. The procedure was realized with a six-needle digital embroidery unit [16] (SWF, Sunstar, Incheon, Korea). Table 1 and Table 2 show the characteristics of the textile electrodes and of the upper and lower commercial threads used in the embroidery patterns, respectively. 

**Figure 3:**
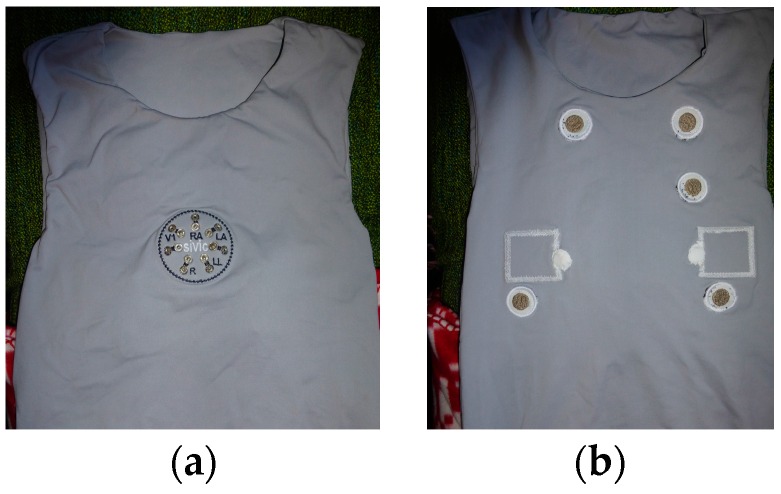
Photos of the T-shirt prototype for cardiorespiratory surveillance; (**a**) outer front-side, and (**b**) inner side.

**Figure 4:**
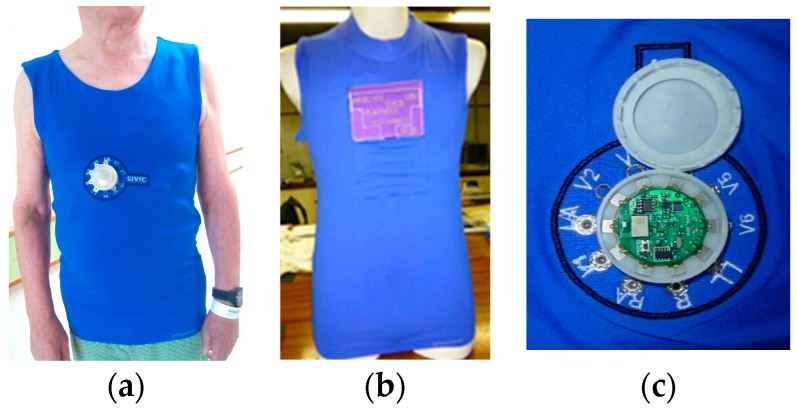
Photos of cardio surveillance T-shirts; (**a**) with embedded DAT unit; (**b**) with SFC for connection to a Holter monitoring device; and (**c**) photo of the DAT unit mounted in a custom designed socket.

The T-shirt system meant for cardiorespiratory surveillance embedded two capacitive textile sensors to capture respiration signals, in addition to five non-removable skin-contact embroidery electrodes, as shown in Figure 3. The garment was conceived for laboratory evaluation of vital signals with an external data acquisition unit. Two cardio surveillance T-shirt prototypes were realized: one embedding a custom DAT unit (Figure 4a) that uses five removable embroidery electrodes, while the other provides snap fasteners for connection to an external Holter monitor (Figure 4b) and integrates five non-removable embroidery electrodes, as requested by the hospital staff, and a pocket to secure the monitoring device. 

The DAT unit, mounted within a custom designed socket fabricated with an additive 3D printing process is also shown in Figure 4c. The socket consists of a base holder and a cap being the DAT board and the power supplying battery (size: 4 × 12 × 29.2 mm) secured in the case by the cap. The socket base holder presents button-like holes through which electrically-conductive sewing pads (4 × 4 mm) provide the attachment to the textile on the outer knit layer of the T-shirt. Furthermore, the soft pads establish electrical contact to the sensors, through the embedded textile cables. Once mounted and secured in the socket with the cap closed, the DAT is electrically connected to the sensors through surface contact of the board pads (5 × 5 mm) to the respective sewing pads. The DAT unit acquires the five-lead ECG signals at a rate of 250 Hz and transmits the compressed data to a smartphone through a Bluetooth low-energy (BLE) link. Further details on the DAT unit are presented in Section 3. 

### 2.2. Experimental Methods and Data Treatment

In all experiments with the T-shirt prototypes, the embroidery electrodes were moist with alcohol prior to the subject’s wearing the systems, in order to expedite the beginning of the data acquisition and to assure identical initial conditions in all tests. 

The cardiorespiratory prototype (Figure 3) was evaluated in laboratory with a subject in a standing state. The seven SFCs of the T-shirt system under test were connected in differential-mode, to a 16-bit programmable data acquisition board NI-DAQ 6212 (National Instruments, Austin, TX, USA), having eight analogue differential inputs with a sensitivity of 6 μV at ±0.2 V and a PC/USB interface. A LabVIEW program (2011 SP1, National Instruments, Austin, TX, USA) controlled the DAQ unit, acquiring the captured signals with a rate of 500 Hz and applied to the ECG and respiratory signals a 50 Hz notch filter. Additionally, the program applied a sixth-order 0.05–100 Hz band-pass Bessel filter to the ECG captured signals. The data were graphically displayed and recorded on a PC. The cardio surveillance prototype, embedding the custom DAT unit (Figure 4), was tested with several subjects in standing and walking conditions. An app designed for an Android smartphone was used to receive, record, and display the ECG raw data. The data recorded in the smartphone were then sent to a PC for further analysis, feature extraction, and data treatment. 

The SNR of the ECG signals recorded by the LabVIEW program and the smartphone app, was calculated using a MATLAB program (R2013a, MathWorks Inc., Natick, MA, USA). The program removes wander interference from the original ECG signals and calculates the SNR ratio in terms of peak-to-peak signal amplitude of the QRS complexes that consist of the Q, R and S waves or deflections and represent the ventricular depolarization of the heart, and baseline noise amplitude. Wander interference originated by respiration activity, present in the raw ECG signals recorded by the smartphone, was removed within the MATLAB program with an equiripple 0.05–100 Hz band-pass digital filter. Baseline wander and baseline drift of lower frequencies (<0.05 Hz) were removed with the program, by processing the waveforms with a smooth function that uses a moving average filter that replaces each data point with the average of the neighbouring data points defined within the span:

(1a) Smooth[y(t), span] = Y(t)

(1b) $$Y\left(t^{i}\right)=\frac{1}{2N+1}\left[y\left(t^{i+N}\right)+y\left(t^{i+N-1}\right)+\ldots +y\left(t^{i-N}\right)\right]$$

where the i index refers to the data point being averaged. A span comprising 200 data entries was used. The program subtracts the smoothed waveform to the original one.

S(t) = y(t) − Y(t)
(1c)

The resulting waveform, S(t), preserves the original peaks of the QRS complex and the original baseline noise amplitude contents. The mean peak-to-peak amplitude value of the QRS complexes, $V^{s}$, and the mean baseline noise amplitude, $V^{n}$, are calculated with the following expressions:

(2a) $$V^{s\;}\;=\;\frac{1}{n}\sum_{i=1}^{n}\left(\left|S_{\max}^{i}{-S}_{\min}^{i}\right|\right)$$

(2b) $$V^{n}\;=\;\frac{1}{n}\sum_{i=1}^{n}\left(\frac{1}{m}\overset{m}{\underset{j=1}{\sum }}2\cdot \left|S^{j}\right|\right)$$

where S_max_, S_min_ correspond to the QRS complex, n is the number of ECG signals in the experimental waveform (n = 20), and m is the number of data points in the baseline portion of each signal (m ≥ 25, depending on the acquisition rate used). The SNR is then given by:

(2c) $$\mathrm{SNR}\;=\;20\cdot \log\frac{V^{s}}{V^{n}}$$

The cardio T-shirt prototype, integrating five non-removable skin-contact textile electrodes and SFC (Figure 2a and Figure 4b), was tested at Hospital da Cova da Beira [29] with ambulatory and internal patients. It was explained to the patients the purpose and terms of the experiments and a consent agreement was signed. The age, height, and weight of the patients were recorded and the body mass index calculated. In each test, the prototype worn by a patient was connected to a three-channel H3 + Holter unit from Mortara (Mortara Instrument, Milwaukee, WI, USA). No skin preparation was applied to the patients. After a quick check on the quality of the signal, the patient was sent away to return in 24 h. The ECG traces were retrieved and analysed at the hospital’s ambulatory office, in a standard manner. The hospital staff in charge of analysing the Holter ECG data was requested to classify the collected signals as true signals (usable signals) or artfacts. The quality of the ECG trace of each patient was evaluated in terms of the duration of time of blocks in which ECG signals were considered usable by the specialists, ${\mathrm{ST}}^{i}$, and given by:

(3) $$\mathrm{usable}\%\;=\;\frac{\sum_{i=1}^{p}{\mathrm{ST}}^{i}}{\mathrm{RT}}\;\times \;100$$

where p is the number of blocks of usable signal and RT is the total duration of the Holter exam.

The prototype was machine washed at ambient temperature with a mild detergent and dried in air, at the hospital, after each ambulatory exam. 

### 2.3. Electrical Characterization 

Rectangular test structures, having lateral dimensions of 50 mm × 5 mm and 120 mm × 6 mm were realized with identical embroidery patterns and materials of the electrodes, for washing and abrasion resistance tests, respectively. The rectangular structures were terminated by circular pads for the measurement of the electrical resistance with the method of two probes, using a Fluke digital multimeter with one digit precision. The resistances of the test structures, R, were measured prior to the laundering/abrasion tests ($R^{0}$) and after the respective test’s cycles ($R^{f}$).

### 2.4. Wear Resistance Tests 

Washing resistance tests were performed with a Linitest machine (Sdl Atlas, Stockport, England) following the ISO 105-C10/2006 guidelines. The tests consisted in laundering test structures and removable embroidery sensors with detergent solution, using 45 min machine washing cycles, at 40 °C, followed by rinsing with water and drying with an air dryer. A total of 30 laundering cycles were carried out to evaluate the sensors’ washing resistance. 

Tests of abrasion were performed with a Mark II tester (Shirley Developments Ltd., Manchester, UK). The test consisted in cycles of abrasion realized by the friction of a wool fabric (abrasion fabric) against the embroidery test structures following a Lissajous pattern of mechanical movement/friction. The tests were run for N abrasion cycles with N = 5000, 10,000, and 30,000.

## 3. ECG Data Acquisition and Transmission Unit

**Figure 5:**
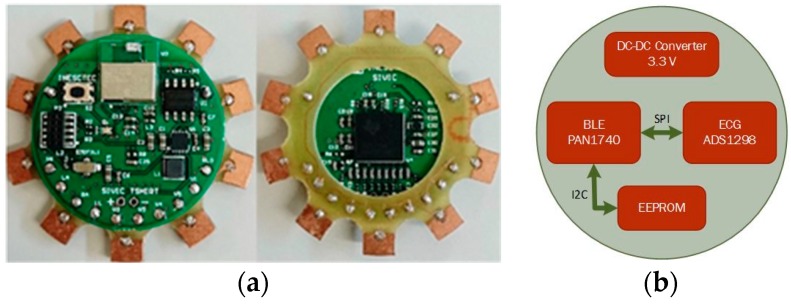
(**a**) Photo of the 12-lead ECG DAT unit (front and back sides); and (**b**) block diagram.

Figure 5 shows the custom 12-lead ECG DAT unit that was developed and the respective block diagram. The number of leads to be used is configurable. It consists of a circular board (30 mm Ø) with an ECG acquisition analogue front-end based on the low-power Texas Instruments 24-bit ADS1298 chip and a PAN1740 BLE module from Panasonic. The board includes also an I2C EEPROM and a DC-DC converter to supply a regulated 3.3 voltage. The PAN1740 is a small (9 mm × 9.5 mm × 1.8 mm) BLE single-mode module based on the Dialog DA14580 SoC with an advertised power consumption of 4.9 mA when transmitting/receiving. This SoC includes a 32-bit ARM Cortex M0 microcontroller (μC, ARM, Cambridge, UK) operating at a 16 MHz frequency, that is used to perform all of the necessary processing operations, thus saving the cost, the additional PCB area, and power consumption of an external μC. The EEPROM was used to save the application code during the development phase. 

The system has three main states of operation: initialization, wait for connection, and running. In the initialization phase the application code is copied from the EEPROM to the DA14580 SRAM and the Bluetooth protocol is initialized; in the wait for connection state the system starts the advertising operation and expects the mobile phone to establish a connection. When this connection is established, the ADS1298 sampling frequency and number of channels are set up. In the running mode, the μC receives a periodic interruption from the ADS1298, indicating the presence of new samples. This initiates a Serial Peripheral Interface (SPI) communication to get the samples from the N channels (N = 3 in the present case) and save them in a buffer. Each T ms, where T can be defined, an application task is executed to check if the buffer size reached 120 bytes. When that is the case, data is copied to the BLE Attribute Protocol (ATT) database and a notification is sent to the mobile phone to initialize the data transmission procedure. The final ECG data processing operations (e.g., decompression, QRS, and T-wave detection algorithms) were implemented in a smartphone, which is, eventually, also responsible to transmit collected data to a healthcare centre for more complex diagnosis. 

**Figure 6:**
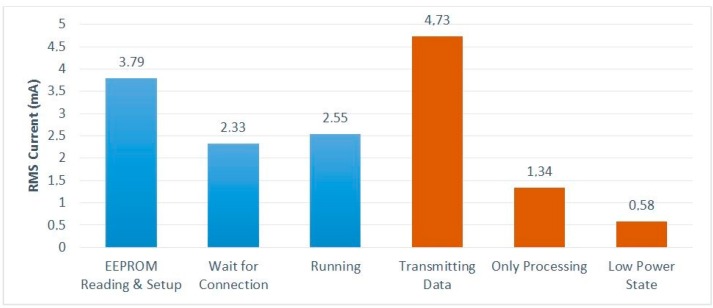
Power consumption of each one of the operation modes and main tasks.

In addition to the selection of low-power devices, we investigated how to minimize the power consumption involved in the data acquisition, processing, and transmission of the captured data. Although 24-bit samples are captured from the ADS1298, only 16-bit resolution samples can be used. This reduction in sample resolution provides a first approach to de-noising and reduction of the power required for transmission. Figure 6 shows, in blue bars, the power supply current consumed by the DAT module in each one of the three modes of operation. The orange bars show the consumption when the three main tasks are performed in isolation, i.e., the same single operation is performed sequentially in a loop. The “Transmitting Data” case gives the consumption measured when data are sent to the smartphone—this consumption is, thus, dominated by the BLE module. It can be seen that, although a low-power BLE link is used, data transmission is still the operation with the highest power consumption—about 3.5 times more than processing. 

The “Running” value in Figure 6 corresponds to the power consumed by the DAT system when performing its mission operation with a T interval of 10 ms. With the 100 mAh battery being used, this consumption provides an autonomy of about 36 h.

## 4. Experimental Results and Discussion

**Figure 7:**
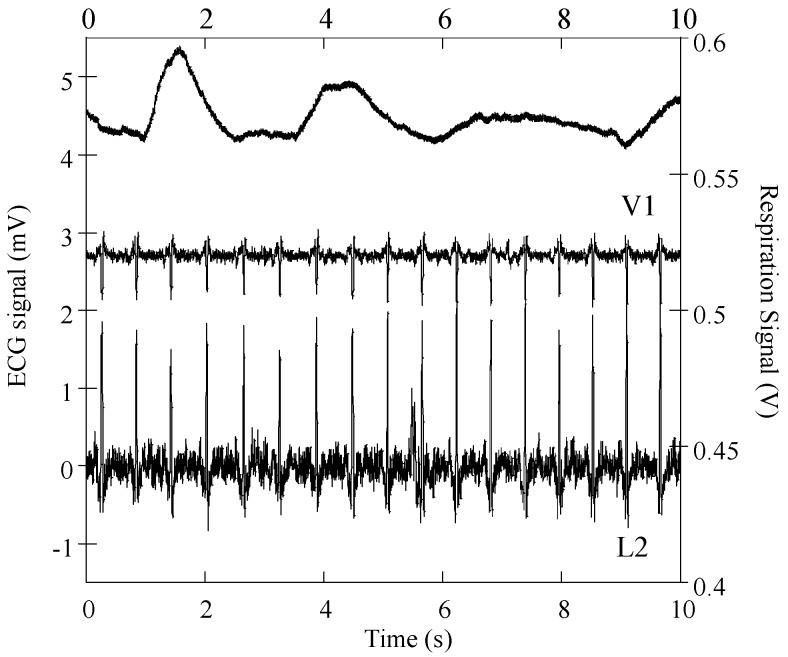
Respiratory and cardiac signals obtained with a subject wearing the cardiorespiratory prototype.

Figure 7 shows ECG (L2 and V1) and respiratory signals obtained in the laboratory with the DAQ NI-6212 unit and a subject wearing the cardiorespiratory T-shirt in a standing position. The ECG and respiratory signals were processed with the MATLAB program and baseline linear drift subtraction, respectively. The graphic shows that derivation L2 has considerably higher noise amplitude than precordial V1, both signals showing SNR amplitudes of 23 dB. This SNR amplitude is considerably lower than that previously obtained with similar embroidery electrodes embedded in the chest band system, on several volunteers [27], with typical values above 30 dB. The high noise amplitude is most likely caused by poor electrical electrode-skin contact in one or both of the electrodes’ placements (RA and/or LL). The electrical electrode-skin contact impedance is the dominant source of noise and is considerably dependent on the pressure applied to the electrodes’ contact region [30,31,32]. This issue was considerably minimized in the second generation of T-shirt bodies, whose construction of tubular knits integrate localized bands of compression. The respiration signals exhibit high sensitivity in accordance with our previous report on chest-band systems [16,33]. The capacitive electrodes exhibit a variation of electrostatic energy accumulated by their conductive surfaces caused by the expansion/contraction of the wearer’s thorax/abdomen. Due to the relatively large surface of the capacitive electrodes, they are prone to signal distortion caused by bending or deformation of the soft textile. 

**Figure 8:**
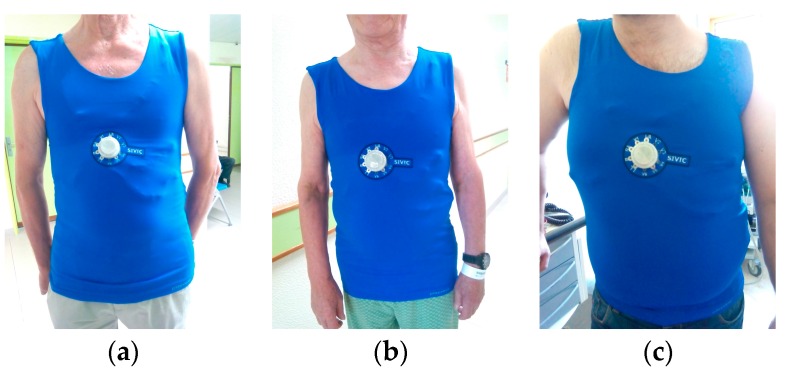
Photos of subjects (**a**–**c**), wearing the cardiac surveillance T-shirt prototype.

**Figure 9:**
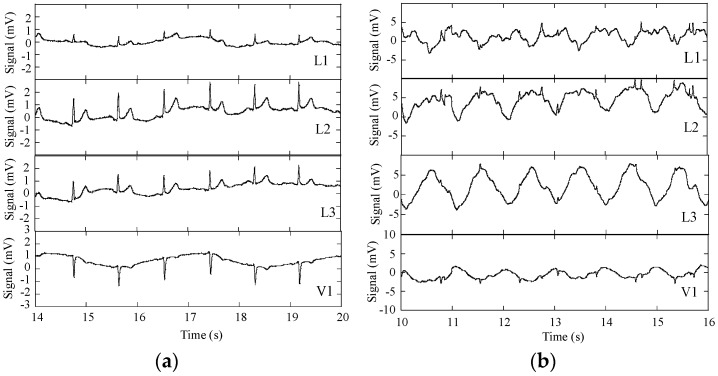
ECG signals L1, L2, L3, and V1 obtained with the subject wearing the cardiac surveillance T-shirt, embedding the DAT unit linked by Bluetooth to a smartphone; (**a**) subject standing; and (**b**) subject walking.

**Figure 10:**
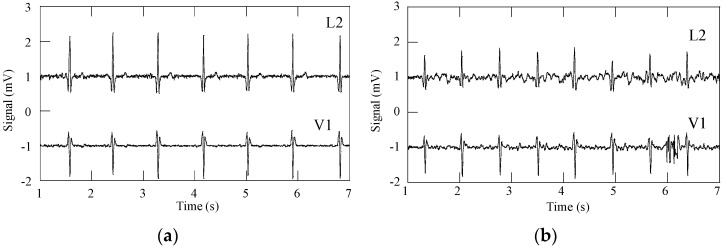
ECG signals L2 and V1, after removal of wander interference: (**a**) subject standing; and (**b**) subject walking.

ECG raw signals, acquired with the DAT unit, were obtained with three subjects wearing the cardio surveillance T-shirt prototype, having distinctive body figures as shown in Figure 8. Figure 9 shows L1, L2, L3, and V1 raw signals as recorded by the smartphone, obtained with subject A (Figure 8) in standing and walking states. In both states of the subject, wander interference caused by the respiration activity is present, most severely in the walking condition. The skin-contact electrodes simultaneously capture the respiratory signals, which can be usable to minimize the number of sensors to integrate in the monitoring garment [34]. The waveforms processed by the MATLAB program (L2 and V1, Figure 10) clearly show that the signals obtained with the subject in the walking state exhibit considerably higher noise amplitude than those corresponding to the standing state.

Table 3 shows the SNR amplitudes corresponding to the ECG signals L2 and V1 obtained with subjects A, B, and C, in standing and walking states. The SNR values clearly indicate that the ECG signals from subjects in the walking state are mostly affected by motion artefact interference, characterized by a decrease of SNR amplitude caused by degradation of electrical electrode-skin contact, with the exception of Subject B. The durations of the tests, of approximately 30 s, are not sufficient to determine whether the T-shirt system better fits Subject B. On the other hand, the results consistently show that the second generation prototype performs reasonably well with the subject steady, characterized by SNR amplitudes in the range of 26 to 37 dB, i.e., of the order of those obtained with gel electrodes [27]. Although gel electrodes also suffer from signal interference due to motion artefacts, they are fixed to the skin through the gel with adhesive properties. The motion artefact interference may be caused by eventual pulling from the cables, but the main source is due to the change of potential across the epidermis during skin deformation by stretching and/or induced by applied pressure [35]. Electrical equivalent models of the electrode-skin interface of gel and textile electrodes have been proposed [31,32,35,36,37]. The gel electrode model includes the contribution of an electrolyte half-cell potential and the epidermis potential. To minimize this interference in the Holter exams with gel electrodes, the skin of the patient is abraded to remove dead cells. Within textile electrodes, the main source of motion artefact interference is caused by the friction of the electrodes on the skin, which causes significant variations in the electrode-skin contact impedance. The amount of pressure applied on the electrode-skin contact surface also affects the electrode’s performance. High pressure lowers the impedance contact and restricts the motion by friction of the electrodes [30,31,36]. 

**Figure 11:**
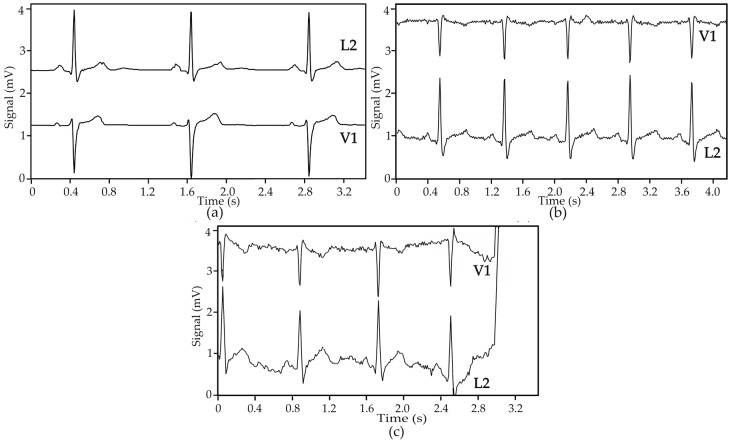
ECG signals obtained in ambulatory with a Holter monitoring device and patient ID = 1: (**a**) gel electrodes, rate 50 BPM; (**b**) textile electrodes, rate 74 BPM; and (**c**) textile electrodes, rate 71 BPM, showing motion artefact interference leading to a loss of signal.

Figure 11 shows ECG signals obtained from two Holter exams realized in a hospital environment with an internal patient; in one exam, the patient was wearing the cardio surveillance T-shirt system, while in the other gel electrodes taped to the thorax were used. In the absence of motion artefact interference, the ECG signals obtained with textile electrodes show slightly higher noise amplitude but comparable quality to that obtained with gel electrodes, as qualified by the hospital’s staff. Poor electrical electrode-skin contact, caused either by the friction of the electrodes against the skin or physical separation between the two, lead to a significant increase of the signals’ source impedance, which is not adapted to the preamplifiers used in Holter monitoring devices, causing interference features similar to that shown in Figure 11c, which may be related to settling time issues induced by large impedance inputs [36,37]. 

Table 4 summarizes the ECG trace quality obtained with five patients wearing the cardio surveillance T-shirt prototype connected to the Holter monitor, where the patient’s ID corresponds to the successive order of the test. The classification (CF) was obtained from an estimation of the patient’s body mass index. The ECG trace quality results show less than 51% of a usable signal, a percentile value considerably lower to the classified as good trace , i.e., with usable percent in the range of 79%–92%. Furthermore, a large variation of ECG trace quality among the patients tested is present, forming two distinctive groups of patients. One group is characterized by usable traces in the range of 30%–50%, (patients ID = 1, 2, and 4), while the other group is characterized by usable traces of the order of less than 5%. However, there is no correlation between the physical parameters and the quality of the traces; e.g., patients 2 and 5, apparently having similar physical characteristics, exhibited 50% and 6% of usable ECG traces, respectively. We think that the abdominal diameter, not registered for the tests, could be an important physical parameter affecting the ECG traces’ results. In the abdominal region, the electrodes R and LL make contact to the patient’s skin, both affecting the measurements of precordial V1 and derivation L2. 

Another explanation for the large disparity between the two groups of patient results can be due to the skin sweat and moisture condition. The patients of the second group may have had a very dry skin condition. When the textile electrodes are dry, they typically exhibit electrical contact impedances with values one to two orders of magnitude higher than that presented by gel electrodes. Several studies in the field describe the importance of moisture in the textile electrodes. It considerably improves the electrode-skin interface, consequently lowering the noise amplitude of the signals to values similar to those of gel electrodes [17,31,38]. Dry electrodes with larger surface tend to exhibit lower noise amplitude and lower contact impedance [38,39]. The materials and physical structure of the electrodes also significantly affect the electrode’s performance [20,27,37,40]. Furthermore, a suitable pre-amplifier can considerably minimize signal interference and propagation of interference in time caused by high impedance input node settling time [33,37]. 

On the other hand, the tests performed with the DAT and in a hospital environment indicated no issues regarding the easiness to find the correct placement of the electrodes, including the precordial V1. 

Table 5 summarizes the results of the resistance to laundering and abrasion tests of the embroidery electrodes, based on the resistance and SNR experimental values, initial ($R^{0}$, ${\mathrm{SNR}}^{0}$*)* and final ($R^{f}$, ${\mathrm{SNR}}^{f}$), obtained with the test structures and embroidery electrodes, respectively. The experimental results show that the embroidery electrodes exhibit good resistance to wear provided by the high compactness and materials of the embroidery patterns.

## 5. Conclusions

This work indicates pathways to achieve high levels of performance in smart garments for future m/pHealth contexts; (1) the use of close-fit elastic garments with a heterogeneous knit structure, integrating specific regions to locally apply pressure over the user’s body while providing a sense of comfort; (2) the use of interchangeable skin-contact electrodes, attached to the clothing by snap fasteners for ease of adaptation of the type of textile electrode to the user; (3) the use of currently available electronic components to realize low-cost ultra-small data acquisition and wireless transmission boards, with low power consumption and the capability to provide the required information in real-time, and linked to popular portable telecommunication devices, such as smartphones and tablets. A novel, simple, and efficient approach to embed the multi-channel DAT unit in the T-shirt system was described.

The heterogeneous tubular knit structures integrating the body of the T-shirt prototypes provided adequate systems performance for subjects in standing states. Motion artefact interference, mainly caused by friction between the textile electrodes and the skin, considerably limited the performance of the prototypes in mobile contexts. The optimization of the location of the electrodes and of the compression bands, as well as the redesign of the textile electrodes should make possible the minimization of the motion artefact interference to acceptable levels. The system’s optimization should be addressed with standardized synchronized measurements of electrical electrode-skin contact impedance and motion on a representative set of subjects such as, e.g., those proposed by Cömert and Hyttinen [35]. Furthermore, the signal processing module should be adapted to textile dry electrodes and cover an adequate range of users’ skin conditions.

The embroidery method is versatile and shows potential to integrate multiple sensors and signal processing modules to realize large surface interactive textile wearable systems, enabling the gathering of large amounts of information, while providing higher security to the user.

## Figures and Tables

**Table 1:** Characteristics of textile materials used in sensors.

Electrode Type	Conductive Textile	*R_s_* (Ω)
Skin contact	Embroidery, textured	0.1
Capacitive	Woven bamboo/30% Ag	<0.1

**Table 2:** Characteristics of thread materials used in the embroidery patterns.

Embroidery Thread	R (Ω·m^−1^)	Manufacturer
Upper	40,000	Less EMF Inc. (Latham, NY, USA)
Upper	200	TibTech (Roncq, France)
Lower	70	Imbut GmbH (Thuringen, Germany)

**Table 3:** SNR of ECG signals recorded with smartphone.

Subject	A		B		C	
ECG signal	L2	V1	L2	V1	L2	V1
SNR (dB) standing sate	34	37	26	32	28	37
SNR (dB) walking state	23	21	23	31	23	24

**Table 4:** ECG trace quality obtained with the cardio surveillance T-shirt prototype.

ID	Age	Weight	Height	CF	RT (min)	Usable %
1	49	83 kg	178 cm	overweight	1427	29
2	76	100 kg	158 cm	obese	1435	50
3	58	81 kg	170 cm	overweight	1440	3
4	39	84 kg	172 cm	overweight	1425	42
5	69	100 kg	176 cm	obese	1409	6

**Table 5:** Wear resistance results.

Test	R^0^ (Ω)	R^f^ (Ω)	SNR^0^ (dB)	SNR^f^ (dB)
laundering	0.7 ± 0.4	0.9 ± 0.4	30.1 ± 0.4 ^1^	29.4 ± 0.1 ^1^
abrasion	1.8 ± 0.4	1.8 ± 0.4	NA	NA

^1^ Tests realized with chest—band system [27].
